# Beyond Neutralizing Antibody Levels: The Epitope Specificity of Antibodies Induced by National Institutes of Health Monovalent Dengue Virus Vaccines

**DOI:** 10.1093/infdis/jiz109

**Published:** 2019-03-21

**Authors:** Jesica A Swanstrom, Usha K Nivarthi, Bhumi Patel, Matthew J Delacruz, Boyd Yount, Douglas G Widman, Anna P Durbin, Stephen S Whitehead, Aravinda M De Silva, Ralph S Baric

**Affiliations:** 1 Department of Epidemiology, Gillings School of Public Health, University of North Carolina at Chapel Hill; 2 Department of Microbiology and Immunology, School of Medicine, University of North Carolina at Chapel Hill; 3 Department of International Health, Johns Hopkins Bloomberg School of Public Health, Baltimore; 4 Center for Immunization Research, Johns Hopkins Bloomberg School of Public Health, Baltimore; 5 Laboratory of Infectious Diseases, National Institute of Allergy and Infectious Diseases, National Institutes of Health, Bethesda, Maryland

**Keywords:** dengue vaccine, flavivirus, neutralizing antibody, live attenuated vaccine

## Abstract

**Background:**

Dengue virus is an emerging mosquito-borne flavivirus responsible for considerable morbidity and mortality worldwide. The Division of Intramural Research, National Institute of Allergy and Infectious Diseases of the US National Institutes of Health (NIH) has developed live attenuated vaccines to each of the 4 serotypes of dengue virus (DENV1–4). While overall levels of DENV neutralizing antibodies (nAbs) in humans have been correlated with protection, these correlations vary depending on DENV serotype, prevaccination immunostatus, age, and study site. By combining both the level and molecular specificity of nAbs to each serotype, it may be possible to develop more robust correlates that predict long-term outcome.

**Methods:**

Using depletions and recombinant chimeric epitope transplant DENVs, we evaluate the molecular specificity and mapped specific epitopes and antigenic regions targeted by vaccine-induced nAbs in volunteers who received the NIH monovalent vaccines against each DENV serotype.

**Results:**

After monovalent vaccination, subjects developed high levels of nAbs that mainly targeted epitopes that are unique (type-specific) to each DENV serotype. The DENV1, 2, and 4 monovalent vaccines induced type-specific nAbs directed to quaternary structure envelope epitopes known to be targets of strongly neutralizing antibodies induced by wild-type DENV infections.

**Conclusions:**

Our results reported here on the molecular specificity of NIH vaccine–induced antibodies enable new strategies, beyond the absolute levels of nAbs, for determining correlates and mechanisms of protective immunity.

## Teaser

National Institutes of Health monovalent dengue vaccines and natural primary dengue virus infections elicit serotype-specific neutralizing antibodies that recognize similar epitopes.

Dengue viruses (DENVs) are mosquito-borne positive-sense RNA viruses in the flavivirus family. The 4 DENV serotypes (DENV1–4) co-circulate in tropical and subtropical regions [1]. A primary infection with a single serotype can be clinically inapparent, present as an acute febrile illness, or, rarely, progress to severe dengue hemorrhagic fever (DHF) [2, 3]. Primary infections stimulate DENV serotype cross-reactive neutralizing antibodies (nAbs), which may provide transient protection against other serotypes, and durable serotype-specific (type-specific) antibodies implicated in long-term protection against reinfection by the homologous serotype [4–6]. Secondary DENV infections with new serotypes also present with symptoms that range from inapparent to severe DHF. However, the risk of DHF is increased in secondary infections, most likely because preexisting poorly neutralizing, cross-reactive antibodies increase the risk of severe disease through a phenomenon known as antibody-dependent enhancement [3]. Hence, leading DENV vaccine formulations are tetravalent to stimulate protective responses to all serotypes and to avoid the possibility of antibody-enhanced disease following vaccination.

The DENV envelope (E) protein is the main target of neutralizing and protective antibodies (Abs) [7–9]. The ectodomain of E is composed of 3 domains: I, II, and III (EDI, EDII, and EDIII). Each DENV virion has 180 monomers of E that are organized into 90 dimers that cover the entire surface of the virus particle. The E proteins are arranged with icosahedral symmetry with each asymmetric unit containing portions of 3 homodimers. Type-specific, neutralizing monoclonal antibodies (MAbs) have been mapped from individuals infected with different DENV serotypes [10–14] that bind to complex quaternary E protein epitopes displayed on intact virions but not on recombinant E protein monomers [10, 11, 15, 16]. Our recent studies have demonstrated that DENV type-specific epitopes defined using human MAbs are also targeted by polyclonal serum nAbs in people exposed to primary infections [10, 11]. In the context of DENV vaccines, type-specific nAbs are likely to be critical for durable protection.

The National Institutes of Health (NIH) tetravalent live attenuated DENV vaccine is composed of DENV1–4 viruses with deletions in the 3′ untranslated region that result in attenuated strains [17–22]. In early-stage human clinical studies, monovalent and tetravalent formulations of the vaccine were immunogenic [19–24]. In a human infection model of DENV2, 1 dose of the tetravalent vaccine provided complete protection against this serotype [25]. The NIH vaccine is currently being evaluated in phase 2 studies in Asia and a large phase 3 efficacy study in Brazil [26]. Recent studies with people exposed to natural DENV infections or live attenuated vaccines [27] indicate that the specificity, rather than total quantity, of nAbs is correlated with long-term protection. We hypothesized that a successful live DENV vaccine should elicit serotype-specific nAbs that are similar to those produced after natural primary infections. Here we report on the molecular specificity of nAbs induced by each component of the NIH vaccine when administered as a monovalent vaccine.

## METHODS

### Subjects and Sera

Healthy volunteers were recruited by the Johns Hopkins Bloomberg School of Public Health Center for Immunization Research (CIR) located in Baltimore, Maryland, to receive one of the monovalent vaccines. The clinical protocol was reviewed and approved by the Joint Committee for Clinical Medicine and informed consent was obtained from each volunteer. Volunteers were recruited and enrolled using previously described eligibility criteria [17–19, 22, 23]. Study subjects for the vaccinations using rDENV1Δ30, rDENV2/4Δ30, rDENV3Δ30/31, and rDENV4Δ30 were enrolled using study protocols CIR-229, CIR-250, CIR-257, and CIR-256, respectively, registered at ClinicalTrials.gov as study numbers NCT00473135 [21], NCT00920517, NCT00831012, and NCT00919178, respectively (https://clinicaltrials.gov). Studies were double-blinded, placebo-controlled phase I trials to evaluate the safety, immunogenicity, and infectivity of low-dose (10 plaque-forming units) live attenuated monovalent DENV vaccines [24]. Serum samples that were collected 6–7.5 months after vaccination with a monovalent vaccine were analyzed in depletion and neutralization studies (Table 1).

**Table 1.** Description of Serum Samples Used for Characterization, Collected 180 or 222 Days Postchallenge

Sample	Sample ID	Days PI	Sample	Sample ID	Days PI
DENV1 monovalent samples	229.01.01	180	DENV2 monovalent samples	250.01.03	180
	229.01.03	180		250.01.07	180
	229.01.05	180		250.01.13	180
	229.01.22	180		250.01.17	180
	229.01.57	180		250.01.24	180
	229.02.39	222		250.01.02	180
	229.02.43	222		250.01.05	180
	229.02.44	222		250.01.10	180
	229.02.49	222		250.01.11	180
	229.02.52	222		250.01.15	180
DENV3 monovalent samples	257.01.01	180	DENV4 monovalent samples	256.01.36	180
	257.01.02	180		256.01.38	180
	257.01.16	180		256.01.46	180
	257.03.49	180		256.01.57	180
	257.03.45	180		256.01.68	180
	257.03.47	180		256.01.51	180
	257.03.50	180		256.01.58	180
				256.01.65	180
				256.01.66	180
				256.01.69	180

Abbreviations: DENV, dengue virus; ID, identifier; PI, postinfection.

### Monovalent DENV Vaccine Strains

Attenuated strains of DENV1 (Westpac 74), and DENV4 (DENV4 virus strain 814669, Dominica, 1981), were achieved by deleting 30 nucleotides from the 3′ untranslated region (UTR), creating rDENV1Δ30 and rDENV4Δ30 [18, 23, 24]. DENV3 (Sleman/78) required 2 separate deletions (30 and 31 nucleotides) in the 3′ UTR for appropriate attenuation for human use DENVΔ30/31 [18, 24, 28]. DENV2/4Δ30 is a chimeric virus in which the DENV2 prM and E genes (New Guinea C [NGC]) replace those of vaccine candidate DENV4Δ30 [19, 24, 29]. Vaccines were produced for human administration using current Good Manufacturing Practices at either Charles River Laboratories (rDENV1Δ30 and rDENV2/4Δ30) or Meridian Life Sciences (rDENV3Δ30/31). L-15 medium (Cambrex Bioscience) was used to dilute the vaccine viruses immediately prior to vaccination. Vaccine virus titers were determined using a standard plaque assay [24].

### Wild-Type and Recombinant DENVs

For laboratory studies, viruses were propagated in C6/36 insect cells or Vero-81 mammalian cells as previously described [30]. DENV1 (American genotype; strain West Pac74), DENV2 (Asian genotype; strain S-16803), DENV3 (Asian genotype; strain CH-53489), and DENV4 (American genotype; strain TVP-376) were used to produce antigen for enzyme-linked immunosorbent assays (ELISAs) and antibody depletion studies. Envelope protein epitope transplant or knockout recombinant DENVs were created using infectious clone systems of the 4 DENV serotypes (DENV1 West Pac 74; DENV2 S-16803; DENV3 Sri Lanka 89; and DENV4 Sri Lanka 92). Recombinant viruses were constructed using the same 4–complementary DNA (cDNA) cloning strategy previously used to create wild-type (WT) DENV infectious clones [11, 30, 31]. The full-length cDNA was transcribed into genome-length RNAs using T7 polymerase. Recombinant genome-length RNA was electroporated into C6/36 cells, and cell culture supernatant containing viable virus was harvested. Recovered virus was sequenced to confirm identity and stored at −80°C. The recombinant viruses used for epitope mapping are listed in Supplementary Table 2.

### Whole Virus Depletion of DENV-Specific Antibodies From Monovalent Vaccine Immune Sera

Purified DENV was absorbed onto 4.5-μm-diameter Polybead polystyrene microspheres (Polysciences, catalog number 17135-5) at a bead (microliters) to ligand (micrograms) ratio of 5:2. Polystyrene beads were washed 3 times with 0.1 M borate buffer (pH 8.5) and incubated with the relevant purified DENV overnight at room temperature. Control beads were incubated overnight with an equivalent amount of bovine serum albumin (BSA). The control and virus-adsorbed beads were blocked with BSA (10 mg/mL)–borate buffer for 30 minutes at room temperature and washed 4 times with phosphate-buffered saline (PBS). DENV-specific antibodies were depleted from human monovalent vaccine sera by incubating virus-adsorbed beads with human sera diluted 1:10 in 1× PBS for 1 hour at 37°C with end-over-end mixing. Samples were subjected to at least 3 sequential rounds of depletions before successful removal of the respective antibodies was confirmed by ELISA.

### ELISA

Initial titration ELISAs were performed to determine the amount of antigen needed to fully deplete the sera from the respective serotype as previously described [27].

### DENV Neutralization Assay

Human sera or MAbs were serially diluted 3-fold and mixed with sufficient virus to cause 15% infection in U937 cells expressing dendritic cell-specific Intercellular adhesion molecule-3-Grabbing Non-Integrin (U937+DC-SIGN) or 50 foci per well in Vero-81 cells. Assays were performed as previously described by our group [32, 33]. Using the 50% neutralization (Neut_50_) titers, the percentage of cross-reactive (CR) and type-specific (TS) nAbs against each serotype were calculated using the following formulas:

$$\%\text{CR Neutralizing Abs}=\;\%\text{TS}\left[\frac{\Delta \;in\;Neut50\;after\;heterologous\;serotype\;depletion}{\Delta \;in\;Neut50\;after\;homologus\;serotype\;depletion}\right]x\;100$$

%TS Neutralizing Abs= 100 − % of CR Abs.

### Statistical Analysis

Nonparametric Kruskal–Wallis Dunn multiple comparisons tests were performed to analyze the differences in mean percentage of type-specific responses and also the mean level of type-specific responses against the 4 serotypes. Similarly, nonparametric Kruskal–Wallis Dunn multiple comparisons test was also used for the epitope mapping data to determine the loss or gain of neutralization to the recombinant chimeric viruses compared to the parental backbone viruses.

## RESULTS

### NIH Monovalent DENV Vaccines Induce Type-Specific nAbs

**Figure 1.**
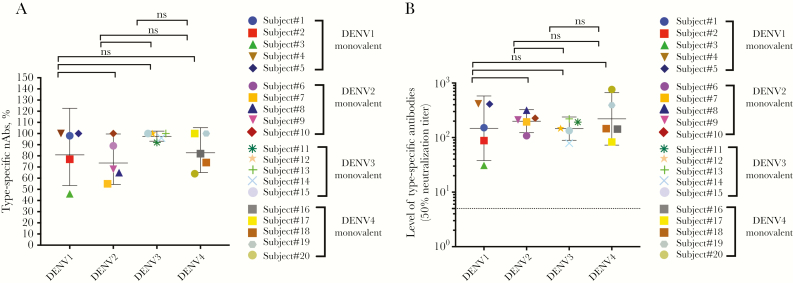
Proportion and level of homotypic (type-specific) neutralizing antibodies (nAbs) to the serotype component in each of the monovalent samples. Antibody depletion assays were used to determine the proportions of dengue virus (DENV) type-specific (homotypic) antibodies induced by the serotype component of the monovalent vaccine. *A*, Each data point depicts the percentage of the total nAb response to the serotype component in the monovalent vaccine attributed to type-specific antibodies. The horizontal bar shows the geometric mean titers and 95% confidence intervals (CIs). There was no significant difference in the mean proportion of type-specific antibodies contributing to neutralization for the serotype component in each monovalent sample. *B*, Each data point represents the absolute level of homotypic nAb to the serotype component in each of the monovalent samples (eg, the 50% neutralization titer against DENV1 in DENV1 monovalent samples). The horizontal bar shows the geometric mean titers and 95% CIs. The dotted line shows the limit of detection value of 5. Abbreviations: DENV, dengue virus; nAbs, neutralizing antibodies; ns, not significant.

We have previously described the overall safety and immunogenicity of each monovalent vaccine component among flavivirus-naive subjects who received a single dose of the vaccine [18, 19, 21–23]. From these studies, we obtained blood from 20 individuals (5 individuals per serotype) 6 months after vaccination to perform antibody depletions and measure levels of serotype-specific nAbs. Polystyrene beads coated with the homotypic (serotype corresponding to the monovalent vaccine) or heterotopic (serotype other than the monovalent vaccine component) purified dengue virions were incubated with the immune sera to deplete specific populations of antibodies as previously described [27]. Following monovalent vaccination, depletion with the homologous serotype results in the removal of all DENV-specific antibody in the sample, whereas depletion with heterologous DENV serotypes results in the removal of serotype cross-reactive antibodies, while retaining type-specific antibodies to the monovalent vaccine component. Antibody depletion against the relevant antigens was confirmed by ELISA before performing DENV neutralization assays (Supplementary Figures 1–4). For example, when a DENV1 monovalent serum sample from subject 229.02.52 was depleted using DENV1 antigen, ELISA confirmed loss of all DENV binding (type-specific and cross-reactive) antibodies and a loss of DENV1 nAbs (Supplementary Figure 1). Depleting DENV1 vaccine sera with heterotypic DENV2 antigen led to the removal of serotype–cross-reactive but not DENV1 type-specific antibodies. DENV1 was efficiently neutralized after removal of cross-reactive antibodies, demonstrating that type-specific antibodies were mainly responsible for neutralization (Supplementary Figure 1). Across the 5 subjects who received the monovalent DENV1 vaccine, on average 84% of DENV1 neutralization was mediated by type-specific antibodies (Figure 1A). Antibody depletion studies also demonstrated that type-specific antibodies were responsible for 75%, 97.6%, and 84% of DENV2, 3, and 4 monovalent vaccine–induced nAb responses, respectively (Figure 1A, and Supplementary Figures 2–4). The net type-specific neutralization titers (Figure 1B) show that all subjects maintained type-specific neutralization titers following depletion.

### Chimeric Recombinant DENV to Map Serotype 1 nAb Responses

**Figure 2.**
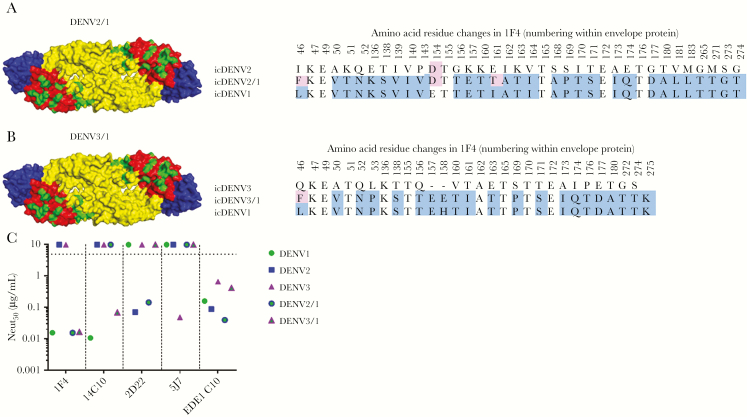
Characterization of dengue virus (DENV) serotype 1, 1F4 recombinant DENV used for mapping the DENV1 responses. Sequence alignments of DENV2/1 (*A*) and DENV3/1 (*B*). *C*, Monoclonal antibody neutralization of DENV2/1 and DENV3/1 compared to each parental strain in the U937+DC-SIGN assay. icDENV indicates DENV infectious clone.

Human monoclonal antibody (hMAb) 1F4 is a type-specific, strongly neutralizing Ab [6] that has been mapped to a complex epitope that includes EDI and the hinge region between EDI and EDII [34]. To measure levels of DENV1 type-specific antibodies directed to this epitope, we constructed recombinant (r) DENV2 (Figure 2A) and DENV3 (Figure 2B) strains displaying the DENV1 1F4 epitope. The 1F4 epitope was transplanted by changing 30 amino acids in DENV2 (Figure 2A) and 21 amino acids in DENV3 (Figure 2B) to DENV1 residues to create rDENV2/1 and rDENV3/1 chimeras. Both rDENV2/1 and rDENV3/1 were bound and neutralized by 1F4 (Figure 2C), demonstrating structural and functional transplantation of the epitope. The hMAb 14C10 is another DENV1 type-specific, strongly neutralizing Ab that binds to a quaternary structure epitope on EDI that overlaps with the 1F4 epitope [10, 13]. The DENV3/1, but not the DENV2/1 chimera, was neutralized by 14C10, demonstrating display of both the 1F4 and 14C10 epitopes on this recombinant virus (Figure 2C). The DENV2/1 strain was neutralized by DENV2 type-specific hMAb 2D22, which has an epitope on EDIII that does not overlap with the footprint of 1F4 (Figure 2C). The DENV3/1 strain was no longer recognized by DENV3 type-specific hMAb 5J7, which has an epitope that overlaps with the 1F4 epitope (Figure 2C). All the WT and recombinant viruses displayed similar neutralization sensitivity to the pan-dengue serotype neutralizing hMAb EDE C10 (Figure 2C), indicating overall preservation of structure between WT and recombinant strains.

### Mapping Type-Specific nAb Responses Induced by Each Monovalent DENV Vaccine

**Figure 3.**
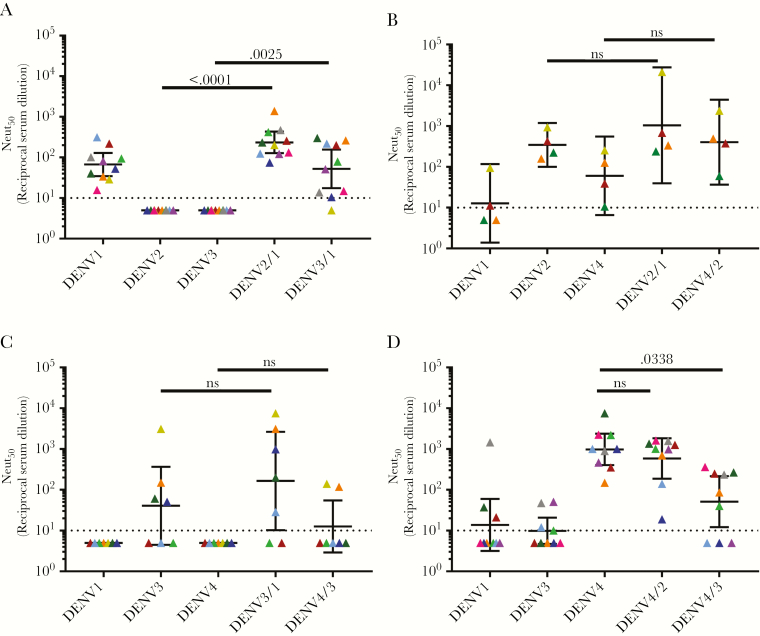
The dengue virus (DENV) serotype 1–4 vaccine strains induce varying levels of type-specific responses against known neutralizing epitopes. Neutralization assays were performed in the U937+DC-SIGN flow-based microneutralization assay. *A*, DENV1 monovalent vaccine. *B*, DENV2 monovalent vaccine. *C*, DENV 3 monovalent vaccine. *D*, DENV4 monovalent vaccine. Each color represents a single sample and the neutralization of each virus (x-axis). Bars indicate the geometric mean titers and 95% confidence intervals of the grouped samples. *P* values are reported between the parental backbone and recombinant DENV strains. Abbreviations: DENV, dengue virus; Neut_50_, 50% neutralization titer; ns, not significant.

Using the panel of WT and epitope transplant recombinant DENVs, we mapped the specificity of type-specific nAbs induced by NIH monovalent vaccines at day 180 or 222 after vaccination (Figure 3, Supplementary Figure 5, and Supplementary Table 1). The neutralization assays with the WT and recombinant DENVs were performed using U937+DC-SIGN cells (Figure 3*A*–*D*) [35] and Vero-81 cells (Supplementary Figure 5*A–D*), which are widely used to evaluate vaccine responses.

In the U937+DC-SIGN neutralization assay, the DENV1 monovalent sera neutralized DENV1 but not DENV2 and DENV3 (Figure 3A). The rDENV2/1 and rDENV3/1 strains were neutralized at levels comparable to WT DENV 1, demonstrating that the region defined by the hMAb 1F4 was a major target of vaccine-induced type-specific nAbs (Figure 3A). The tracking of DENV1 nAbs was more consistent across individuals with the rDENV2/1 strain compared with the rDENV3/1 strain, demonstrating the impact of the backbone sequence on epitope display (Figure 3A). In the Vero-81 cell–based neutralization assay, the DENV1 monovalent vaccine sera efficiently neutralized DENV1 and demonstrated low-level cross-neutralization of DENV2 and DENV3 (Supplementary Figure 5*A*). Moreover, in this assay we observed no evidence for epitope-specific tracking with the rDENV2/1 or rDENV3/1 strains, although there was a trend toward tracking with rDENV2/1 that did not reach statistical significance (Supplementary Figure 5*A*).

The subjects who received the monovalent DENV2 vaccine had high levels of nAbs to DENV2 and low levels of cross-neutralizing Abs to DENV1 and DENV4 with both cell lines (Figure 3*B* and Supplementary Figure 5*B*). The recombinant DENV2/1 strain was neutralized at levels comparable to the WT DENV2 strain, indicating that EDI was not the main target of DENV2 vaccine–induced nAbs (Figure 3B and Supplementary Figure 5*B*). The recombinant DENV4/2 strain containing EDIII from DENV2 was efficiently neutralized by subjects who received the DENV2 vaccine in the Vero-81 cell–based assay (Supplementary Figure 5*B*), confirming our previous observations that the DENV2-induced nAbs targeted type-specific epitopes centered on EDIII [11]. Due to limited availability of sample volume, we were only able to test 4 monovalent DENV2 samples in the U937+DC-SIGN assay (Figure 3B). While we observed a trend of DENV2 nAb tracking with the rDENV4/2 virus with these cells, the difference did not reach statistical significance (Figure 3B).

The subjects who received the monovalent DENV3 vaccine had the highest levels of nAbs to this serotype and low levels of cross-neutralizing antibodies to DENV1 or DENV4 depending on the cell type used for the assay (Figure 3C and Supplementary Figure 5*C*). No appreciable loss of DENV3 neutralization was observed with the DENV3/1 virus, suggesting that this region was not a major target of vaccine-induced DENV3 nAbs. The previously described rDENV4/3, displaying the DENV3 type-specific, strongly neutralizing hMAb 5J7 quaternary structure epitope centered on the hinge region between EDI and EDII [36, 37], was not significantly neutralized by vaccine immune sera, demonstrating that the transplanted epitope was not a major target of type-specific nAbs (Figure 3C and Supplementary Figure 5*C*). We noted a modest gain of DENV3 nAbs in 2 subjects, indicating that the DENV3 vaccine can induce low levels of antibodies that track with this epitope in some people (Figure 3C).

When mapping the DENV4 monovalent vaccine–induced response, we used the DENV4/2 and DENV4/3 (Supplementary Table 1) loss-of-function mutants. Subjects who received the DENV4 vaccine strongly neutralized DENV4 and displayed low to no cross-neutralizing antibodies to DENV2 and DENV3 in both assays (Figure 3D and Supplementary Figure 5*D*). The recombinant DENV4/2 strain was efficiently neutralized by the vaccine sera at levels comparable to WT DENV4, indicating that EDIII was not a major target of DENV4 vaccine–induced nAbs (Figure 3D and Supplementary Figure 5*D*). DENV4 vaccine sera poorly neutralized the recombinant DENV4/3 strain compared to the parental DENV4 (Figure 3D) in the U937+DC-SIGN cell assay, indicating that some DENV4 nAbs target epitopes near the hinge between EDI and EDII. The loss of DENV4 neutralization was not observed when the DENV4/3 strain was used in the Vero cell assay, indicating the importance of tracking epitope specific responses with different cell lines.

## DISCUSSION

The leading tetravalent live attenuated DENV vaccines aim to stimulate balanced protective immune responses to all 4 DENV serotypes simultaneously. The preclinical and early clinical development of these vaccines has been largely guided by the ability of vaccines to induce nAbs to each DENV serotype because nAbs have been correlated with vaccine efficacy [25, 27, 38]. However, the first tetravalent live DENV vaccine to complete phase 3 efficacy trials has demonstrated that the mere presence of nAb to a particular serotype is not sufficient to protect people from infection and disease [27]. Data from vaccine trials and other studies point to both the level and quality of nAbs playing a role in durable protective immunity [27]. Among people exposed to primary DENV infections, nAbs targeting serotype-specific epitopes are strongly correlated with protection against the homologous serotype [39]. Herein, we evaluated the quality of nAbs induced by the NIH monovalent vaccine components in dengue-naive individuals.

Flavivirus-naive subjects who received each monovalent vaccine developed high levels of nAbs to the vaccine-matched serotype and lower levels to the other serotypes. Antibody depletion assays established that 70%–90% of nAb response was directed to type-specific epitopes on the vaccine-matched serotype. The DENV3 monovalent vaccine virus elicited the highest proportion of type-specific neutralizing responses, followed by equal proportions elicited by the DENV1 and DENV4 monovalent vaccines; the lowest proportion of type-specific antibodies was elicited by the DENV2 monovalent vaccine. Having established that each vaccine component is capable of eliciting a type-specific neutralizing response, it will be interesting and important to evaluate if these type-specific responses are preserved when the 4 components are injected as a tetravalent formulation.

We determined if vaccine-induced type-specific antibodies recognized known immunodominant nAb epitopes on each serotype. This required the assembly of 2 novel recombinant chimeric viruses, DENV2/1 and DENV3/1, that display the DENV1 1F4 neutralizing epitope on a heterologous serotype backbone. While both recombinant viruses were recognized by hMAb 1F4, the DENV3/1 chimera was also recognized by the DENV1-specific hMAb 14C10, which has an epitope that overlaps with 1F4. The DENV3/1 chimera was not neutralized by DENV3 type-specific hMAb 5J7, indicating that the slight overlap between the DENV1 1F4 and DENV3 5J7 epitopes resulted in the disruption of the 5J7 epitope. The DENV1 monovalent vaccine–induced antibody response that tracked with the 1F4 epitope displayed on both DENV2/1 and 3/1 viruses in the neutralization assay with U937+DC-SIGN–expressing cells and also displayed a trend of tracking with the 1F4 epitope in the Vero cell–based assay.

The type-specific nAbs induced by the DENV2 monovalent vaccine tracked well with the recombinant DENV4/2 EDIII chimera, confirming our previous observations that the vaccine induces a response that tracks with EDIII [32].

The hMAb 5J7 is the only DENV3 type-specific human nAb that has been characterized to date [6, 12]. The DENV3 vaccine type-specific responses did not track with this epitope in both gain-of-function (DENV4/3 virus displaying 5J7 epitope) or loss-of-function (DENV3/1 virus) neutralization assays. Our results indicate that other epitopes, yet to be fully described, are the major targets of the DENV3 type-specific response. Indeed, recent studies demonstrate that the 5J7 epitope is not the major target of nAbs in people exposed to primary DENV3 infections as well [36].

The DENV4 monovalent vaccine response appears to target epitopes centered near the hinge region between EDI/EDII because the DENV4/3 chimera was poorly neutralized compared to WT DENV4 in the neutralization assay using U937+DC-SIGN cells. Indeed, 2 strongly neutralizing hMAbs (DV4-126 and DV4-131) isolated from individuals infected with DENV4 have been mapped to this region on DENV4, implicated as a target for vaccine-induced nAbs [14].

We used both Vero and U937+DC-SIGN cells to map vaccine-induced nAb responses because of recent data from vaccine trials indicating that titers measured with the Vero cell assay may not be sufficient for protection [18]. Indeed, we observed interesting differences between Vero and U937+DC-SIGN cells with monovalent vaccine immune sera. The DENV1 and DENV4 monovalent vaccine responses tracked with known epitopes on EDI and the EDI/EDII hinge region in the U937+DC-SIGN assay but not the traditional Vero cell–based assay. Numerous factors may be contributing to this discordance, noting that the entry receptor for Vero cells remains unknown, but is thought to engage the E glycoprotein EDIII region, whereas DC-SIGN–mediated entry is via binding to the *N*-linked glycan on EDII [40]. In addition, as some antibodies can bind to DENVs and promote Fc receptor mediated entry and infection of cells, the presence of Fc receptors on U937 but not Vero cells may also account for the observed differences. We recently observed that DENV1 strains circulating in people are fully mature, whereas the same strains passaged on laboratory cell lines are less efficiently processed and released as partially mature virions [41]. Differences in the maturation state of DENVs can also alter antibody neutralization in a cell type–dependent manner [42].

Overall, this study continues to apply new pioneering approaches and assays to evaluate the quality of nAbs elicited by individual components of a live attenuated DENV tetravalent vaccine. More significantly, we also mapped vaccine type–specific neutralizing responses to epitopes that are also targeted during natural infections. Importantly, these studies provide valuable insights about the quality of the nAb responses elicited by the monovalent vaccines in isolation. Although these data predict that each individual component is primed to elicit robust type-specific responses, comparative studies in volunteers receiving the tetravalent vaccine will identify whether certain components elicit immunodominant epitope-specific or balanced responses across all 4 components. Large-sized human clinical efficacy trials will actually show if these qualitative trends are also reflected on a larger population in endemic regions receiving tetravalent vaccines.

## Supplementary Data

Supplementary materials are available at *The Journal of Infectious Diseases* online. Consisting of data provided by the authors to benefit the reader, the posted materials are not copyedited and are the sole responsibility of the authors, so questions or comments should be addressed to the corresponding author.

jiz109_suppl_Supplementary_Figure_S1

jiz109_suppl_Supplementary_Figure_S2

jiz109_suppl_Supplementary_Figure_S3

jiz109_suppl_Supplementary_Figure_S4

jiz109_suppl_Supplementary_Figure_S5

jiz109_suppl_Supplementary_Table_S1

jiz109_suppl_Supplementary_Table_S2

## Notes

***Financial support.* **Funding for this work was provided by the Bill & Melinda Gates Foundation and the NIH/National Institute of Allergy and Infectious Diseases (grant numbers AI107731 and AI106695).

***Potential conflicts of interest.* **A. M. S. has consulted on dengue vaccines for Takeda Vaccines, Sanofi Pasteur, GSK, and Merck Pharmaceuticals and is also an inventor in patents related to dengue vaccines. R. S. B. has consulted on dengue vaccines for Takeda Vaccines and Sanofi Pasteur and is also an inventor in patents related to dengue vaccines. All other authors report no potential conflicts of interest. All authors have submitted the ICMJE Form for Disclosure of Potential Conflicts of Interest. Conflicts that the editors consider relevant to the content of the manuscript have been disclosed.

Presented in part: American Society of Tropical Medicine and Hygiene Annual Meeting, Baltimore, Maryland, 5–9 November 2017. Oral presentation 73.

## References

[1] Bhatt S, Gething PW, Brady OJ, et al. The global distribution and burden of dengue. Nature 2013; 496:504–7. 23563266 10.1038/nature12060 PMC3651993

[2] Burke DS, Nisalak A, Johnson DE, Scott RM A prospective study of dengue infections in Bangkok. Am J Trop Med Hyg 1988; 38:172–80. 3341519 10.4269/ajtmh.1988.38.172

[3] de Alwis R, Williams KL, Schmid MA, et al. Dengue viruses are enhanced by distinct populations of serotype cross-reactive antibodies in human immune sera. PLoS Pathog 2014; 10:e1004386. 25275316 10.1371/journal.ppat.1004386 PMC4183589

[4] Wahala WM, Silva AM The human antibody response to dengue virus infection. Viruses 2011; 3:2374–95. 22355444 10.3390/v3122374 PMC3280510

[5] Clapham HE, Rodriguez-Barraquer I, Azman AS, et al. Dengue virus (DENV) neutralizing antibody kinetics in children after symptomatic primary and postprimary DENV infection. J Infect Dis 2016; 213:1428–35. 26704615 10.1093/infdis/jiv759 PMC4813744

[6] de Alwis R, Smith SA, Olivarez NP, et al. Identification of human neutralizing antibodies that bind to complex epitopes on dengue virions. Proc Natl Acad Sci U S A 2012; 109:7439–44. 22499787 10.1073/pnas.1200566109 PMC3358852

[7] Smith SA, de Alwis AR, Kose N, Jadi RS, de Silva AM, Crowe JE Jr Isolation of dengue virus-specific memory B cells with live virus antigen from human subjects following natural infection reveals the presence of diverse novel functional groups of antibody clones. J Virol 2014; 88:12233–41. 25100837 10.1128/JVI.00247-14 PMC4248927

[8] Smith SA, de Alwis R, Kose N, et al. Human monoclonal antibodies derived from memory B cells following live attenuated dengue virus vaccination or natural infection exhibit similar characteristics. J Infect Dis 2013; 207:1898–908. 23526830 10.1093/infdis/jit119 PMC3654755

[9] Beltramello M, Williams KL, Simmons CP, et al. The human immune response to dengue virus is dominated by highly cross-reactive antibodies endowed with neutralizing and enhancing activity. Cell Host Microbe 2010; 8:271–83. 20833378 10.1016/j.chom.2010.08.007 PMC3884547

[10] Fibriansah G, Tan JL, Smith SA, et al. A potent anti-dengue human antibody preferentially recognizes the conformation of E protein monomers assembled on the virus surface. EMBO Mol Med 2014; 6:358–71. 24421336 10.1002/emmm.201303404 PMC3958310

[11] Gallichotte EN, Widman DG, Yount BL, et al. A new quaternary structure epitope on dengue virus serotype 2 is the target of durable type-specific neutralizing antibodies. MBio 2015; 6:e01461–15. 26463165 10.1128/mBio.01461-15 PMC4620467

[12] Fibriansah G, Tan JL, Smith SA, et al. A highly potent human antibody neutralizes dengue virus serotype 3 by binding across three surface proteins. Nat Commun 2015; 6:6341. 25698059 10.1038/ncomms7341 PMC4346626

[13] Teoh EP, Kukkaro P, Teo EW, et al. The structural basis for serotype-specific neutralization of dengue virus by a human antibody. Sci Transl Med 2012; 4:139ra83. 10.1126/scitranslmed.3003888 22723463

[14] Nivarthi UK, Kose N, Sapparapu G, et al Mapping the human memory B cell and serum neutralizing antibody responses to dengue virus serotype 4 infection and vaccination. J Virol 2017; 91. doi:10.1128/JVI.02041-16. PMC5309932 28031369

[15] Chaudhury S, Gromowski GD, Ripoll DR, Khavrutskii IV, Desai V, Wallqvist A Dengue virus antibody database: systematically linking serotype-specificity with epitope mapping in dengue virus. PLoS Negl Trop Dis 2017; 11:e0005395. 28222130 10.1371/journal.pntd.0005395 PMC5336305

[16] Fibriansah G, Ibarra KD, Ng TS, et al. Dengue virus. Cryo-EM structure of an antibody that neutralizes dengue virus type 2 by locking E protein dimers. Science 2015; 349:88–91. 26138979 10.1126/science.aaa8651 PMC4672004

[17] Durbin AP, Karron RA, Sun W, et al. Attenuation and immunogenicity in humans of a live dengue virus type-4 vaccine candidate with a 30 nucleotide deletion in its 3’-untranslated region. Am J Trop Med Hyg 2001; 65:405–13. 11716091 10.4269/ajtmh.2001.65.405

[18] Durbin AP, Kirkpatrick BD, Pierce KK, Schmidt AC, Whitehead SS Development and clinical evaluation of multiple investigational monovalent DENV vaccines to identify components for inclusion in a live attenuated tetravalent DENV vaccine. Vaccine 2011; 29:7242–50. 21781997 10.1016/j.vaccine.2011.07.023 PMC3170437

[19] Durbin AP, McArthur JH, Marron JA, et al. rDEN2/4Delta30(ME), a live attenuated chimeric dengue serotype 2 vaccine is safe and highly immunogenic in healthy dengue-naïve adults. Hum Vaccin 2006; 2:255–60. 17106267 10.4161/hv.2.6.3494

[20] Durbin AP, Kirkpatrick BD, Pierce KK, et al. A single dose of any of four different live attenuated tetravalent dengue vaccines is safe and immunogenic in flavivirus-naive adults: a randomized, double-blind clinical trial. J Infect Dis 2013; 207:957–65. 23329850 10.1093/infdis/jis936 PMC3571448

[21] Durbin AP, Whitehead SS, Shaffer D, et al. A single dose of the DENV-1 candidate vaccine rDEN1Δ30 is strongly immunogenic and induces resistance to a second dose in a randomized trial. PLoS Negl Trop Dis 2011; 5:e1267. 21829748 10.1371/journal.pntd.0001267 PMC3149013

[22] Durbin AP, Whitehead SS, McArthur J, et al. rDEN4delta30, a live attenuated dengue virus type 4 vaccine candidate, is safe, immunogenic, and highly infectious in healthy adult volunteers. J Infect Dis 2005; 191:710–8. 15688284 10.1086/427780

[23] Durbin AP, McArthur J, Marron JA, et al. The live attenuated dengue serotype 1 vaccine rDEN1Delta30 is safe and highly immunogenic in healthy adult volunteers. Hum Vaccin 2006; 2:167–73. 17012875 10.4161/hv.2.4.2944

[24] Lindow JC, Durbin AP, Whitehead SS, Pierce KK, Carmolli MP, Kirkpatrick BD Vaccination of volunteers with low-dose, live-attenuated, dengue viruses leads to serotype-specific immunologic and virologic profiles. Vaccine 2013; 31:3347–52. 23735680 10.1016/j.vaccine.2013.05.075 PMC3777849

[25] Kirkpatrick BD, Whitehead SS, Pierce KK, et al. The live attenuated dengue vaccine TV003 elicits complete protection against dengue in a human challenge model. Sci Transl Med 2016; 8:330ra36. 10.1126/scitranslmed.aaf1517 27089205

[26] Whitehead SS, Durbin AP, Pierce KK, et al. In a randomized trial, the live attenuated tetravalent dengue vaccine TV003 is well-tolerated and highly immunogenic in subjects with flavivirus exposure prior to vaccination. PLoS Negl Trop Dis 2017; 11:e0005584. 28481883 10.1371/journal.pntd.0005584 PMC5436874

[27] Henein S, Swanstrom J, Byers AM, et al. Dissecting antibodies induced by a chimeric yellow fever-dengue, live-attenuated, tetravalent dengue vaccine (CYD-TDV) in naive and dengue-exposed individuals. J Infect Dis 2017; 215:351–8. 27932620 10.1093/infdis/jiw576 PMC6392503

[28] Blaney JE Jr, Sathe NS, Goddard L, et al. Dengue virus type 3 vaccine candidates generated by introduction of deletions in the 3’ untranslated region (3’-UTR) or by exchange of the DENV-3 3’-UTR with that of DENV-4. Vaccine 2008; 26:817–28. 18191005 10.1016/j.vaccine.2007.11.082 PMC2246307

[29] Whitehead SS, Hanley KA, Blaney JE Jr, Gilmore LE, Elkins WR, Murphy BR Substitution of the structural genes of dengue virus type 4 with those of type 2 results in chimeric vaccine candidates which are attenuated for mosquitoes, mice, and rhesus monkeys. Vaccine 2003; 21:4307–16. 14505913 10.1016/s0264-410x(03)00488-2

[30] Messer WB, Yount B, Hacker KE, et al. Development and characterization of a reverse genetic system for studying dengue virus serotype 3 strain variation and neutralization. PLoS Negl Trop Dis 2012; 6:e1486. 22389731 10.1371/journal.pntd.0001486 PMC3289595

[31] Messer WB, Yount BL, Royal SR, et al. Functional transplant of a dengue virus serotype 3 (DENV3)-specific human monoclonal antibody epitope into DENV1. J Virol 2016; 90:5090–7. 26962223 10.1128/JVI.00155-16 PMC4859728

[32] Gallichotte EN, Baric TJ, Yount BL Jr, et al. Human dengue virus serotype 2 neutralizing antibodies target two distinct quaternary epitopes. PLoS Pathog 2018; 14:e1006934. 29481552 10.1371/journal.ppat.1006934 PMC5843351

[33] Swanstrom JA, Henein S, Plante JA, et al. Analyzing the human serum antibody responses to a live attenuated tetravalent dengue vaccine candidate. J Infect Dis 2018; 217:1932–41. 29800370 10.1093/infdis/jiy063 PMC5972589

[34] Fibriansah G, Tan JL, Smith SA, et al. A potent anti-dengue human antibody preferentially recognizes the conformation of E protein monomers assembled on the virus surface. EMBO Mol Med 2014; 6:358–71. 24421336 10.1002/emmm.201303404 PMC3958310

[35] Kraus AA, Messer W, Haymore LB, de Silva AM Comparison of plaque- and flow cytometry-based methods for measuring dengue virus neutralization. J Clin Microbiol 2007; 45:3777–80. 17804661 10.1128/JCM.00827-07 PMC2168473

[36] Andrade DV, Katzelnick LC, Widman DG, et al Analysis of individuals from a dengue-endemic region helps define the footprint and repertoire of antibodies targeting dengue virus 3 type-specific epitopes. MBio 2017; 8. doi:10.1128/mBio.01205-17. PMC5605938 28928210

[37] Widman DG, Young E, Nivarthi U, et al. Transplantation of a quaternary structure neutralizing antibody epitope from dengue virus serotype 3 into serotype 4. Sci Rep 2017; 7:17169. 29215033 10.1038/s41598-017-17355-5 PMC5719398

[38] Osorio JE, Wallace D, Stinchcomb DT A recombinant, chimeric tetravalent dengue vaccine candidate based on a dengue virus serotype 2 backbone. Expert Rev Vaccines 2016; 15:497–508. 26635182 10.1586/14760584.2016.1128328

[39] Patel B, Longo P, Miley MJ, Montoya M, Harris E, de Silva AM Dissecting the human serum antibody response to secondary dengue virus infections. PLoS Negl Trop Dis 2017; 11:e0005554. 28505154 10.1371/journal.pntd.0005554 PMC5444852

[40] Shah M, Wadood A, Rahman Z, Husnain T Interaction and inhibition of dengue envelope glycoprotein with mammalian receptor DC-sign: an in-silico approach. PLoS One 2013; 8:e59211. 23527139 10.1371/journal.pone.0059211 PMC3601059

[41] Raut R, Corbett KS, Tennekoon RN, et al. Dengue type 1 viruses circulating in humans are highly infectious and poorly neutralized by human antibodies. Proc Natl Acad Sci U S A 2019; 116:227–32. 30518559 10.1073/pnas.1812055115 PMC6320508

[42] Mukherjee S, Dowd KA, Manhart CJ, et al. Mechanism and significance of cell type-dependent neutralization of flaviviruses. J Virol 2014; 88:7210–20. 24741083 10.1128/JVI.03690-13 PMC4054442

