# Supplementary figures and images for: Beyond Neutralizing Antibody Levels: The Epitope Specificity of Antibodies Induced by National Institutes of Health Monovalent Dengue Virus Vaccines
Source: J Infect Dis. 2019 Mar 21;220(2):219–27. doi: 10.1093/infdis/jiz109 (PMC6581895; doi:10.1093/infdis/jiz109)

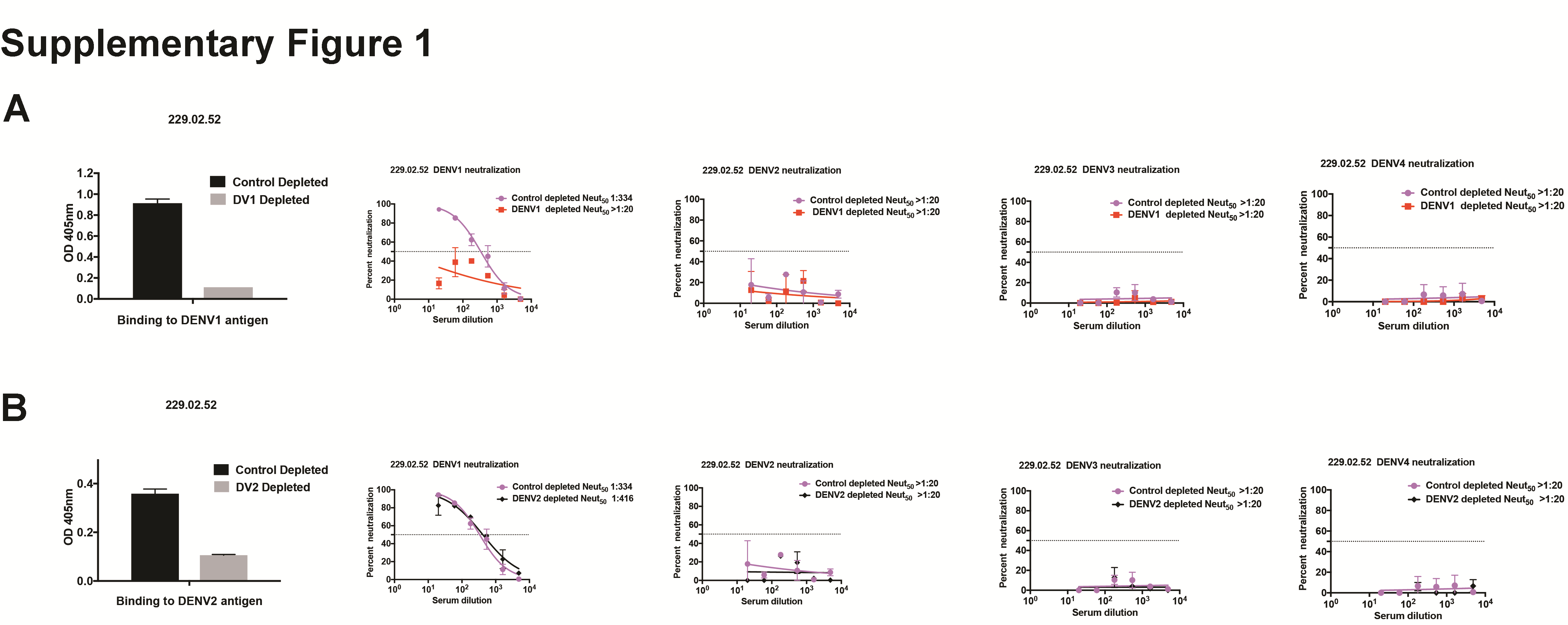

Supplement: jiz109_suppl_Supplementary_Figure_S1 [file jiz109_suppl_supplementary_figure_s1.png]

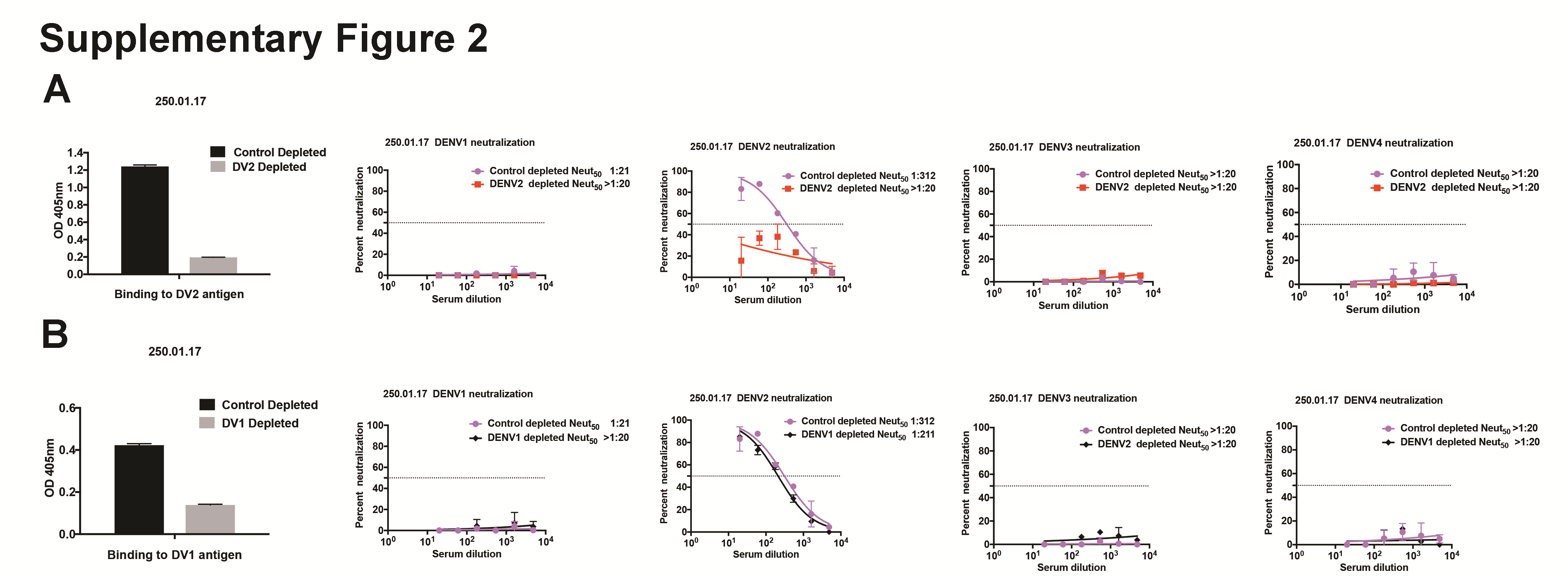

Supplement: jiz109_suppl_Supplementary_Figure_S2 [file jiz109_suppl_supplementary_figure_s2.png]

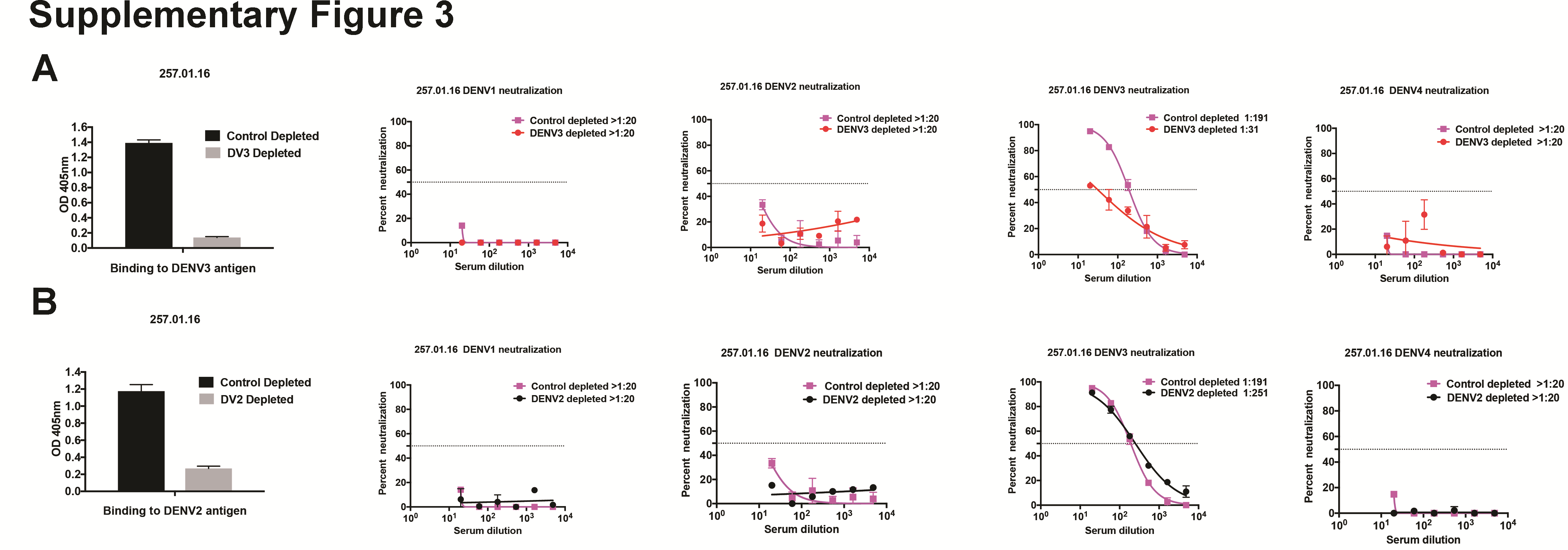

Supplement: jiz109_suppl_Supplementary_Figure_S3 [file jiz109_suppl_supplementary_figure_s3.png]

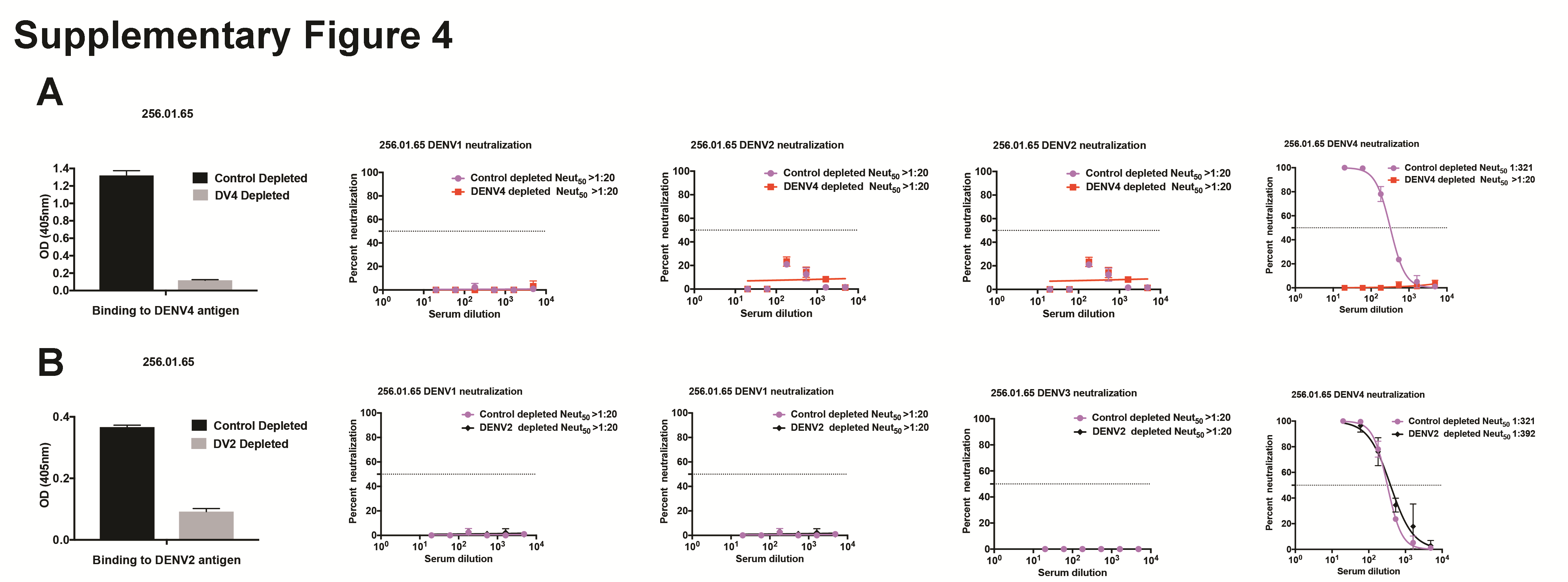

Supplement: jiz109_suppl_Supplementary_Figure_S4 [file jiz109_suppl_supplementary_figure_s4.png]

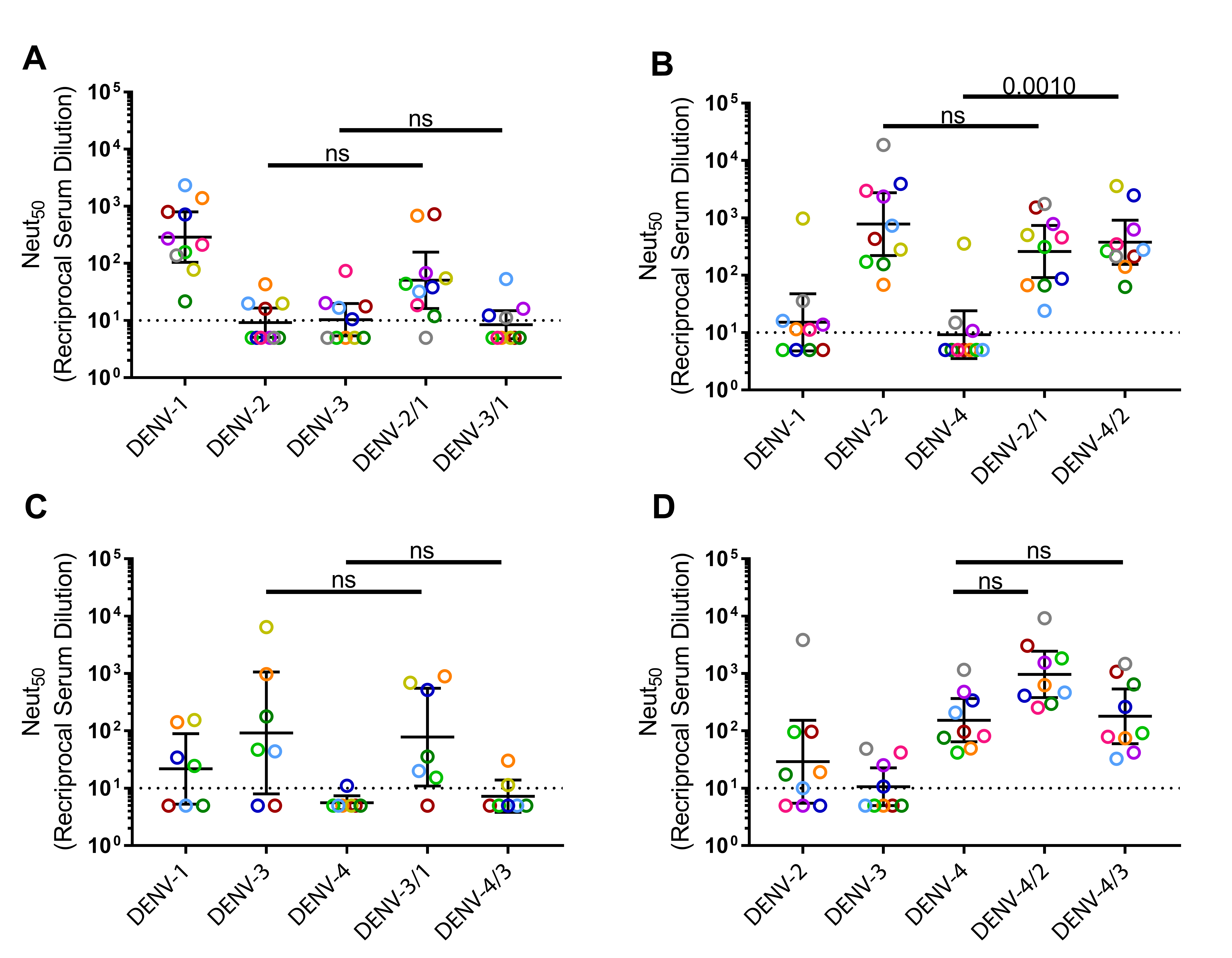

Supplement: jiz109_suppl_Supplementary_Figure_S5 [file jiz109_suppl_supplementary_figure_s5.png]
