# Supplementary material for: Beyond Neutralizing Antibody Levels: The Epitope Specificity of Antibodies Induced by National Institutes of Health Monovalent Dengue Virus Vaccines
Source: J Infect Dis. 2019 Mar 21;220(2):219–27. doi: 10.1093/infdis/jiz109 (PMC6581895; doi:10.1093/infdis/jiz109)
Supplement: jiz109_suppl_Supplementary_Table_S1 [file jiz109_suppl_supplementary_table_s1.docx]

**Table S1.** Panel of monovalent sera with neutralization titers following heterotypic neutralization

| Sera | Sample ID | BSA Depleted (Neut_50_) | Heterotypic Depleted (Neut_50_) | % loss of Neutralization | % Type -specific neutralizing antibodies |
| --- | --- | --- | --- | --- | --- |
| DENV1 immune sera | Subject #1 | 155 | 152 | 2% | 98% |
|  | Subject #2 | 114 | 88 | 23% | 77% |
|  | Subject #3 | 68 | 31 | 54% | 46% |
|  | Subject #4 | 334 | 416 | 0% | 100% |
|  | Subject #5 | 249 | 411 | 0% | 100% |
|  | *Average* |  |  | *16%* | *84%* |
| DENV2 immune sera | Subject #6 | 121 | 108 | 11% | 89% |
|  | Subject #7 | 350 | 194 | 45% | 55% |
|  | Subject #8 | 491 | 318 | 35% | 65% |
|  | Subject #9 | 312 | 211 | 32% | 68% |
|  | Subject #10 | 210 | 227 | 0% | 100% |
|  | *Average* |  |  | *25%* | *75%* |
| DENV3 immune sera | Subject #11 | 208 | 192 | 8% | 92% |
|  | Subject #12 | 103 | 148 | 0% | 100% |
|  | Subject #13 | 191 | 220 | 0% | 100% |
|  | Subject #14 | 82 | 79 | 4% | 96% |
|  | Subject #15 | 109 | 134 | 0% | 100% |
|  | *Average* |  |  | *2.4%* | *97.6%* |
| DENV4 Immune sera | Subject #16 | 175 | 143 | 18% | 82% |
|  | Subject #17 | 61 | 83 | 0% | 100% |
|  | Subject #18 | 198 | 146 | 26% | 74% |
|  | Subject #19 | 321 | 392 | 0% | 100% |
|  | Subject #20 | 1196 | 762 | 36% | 64% |
|  | *Average* |  |  | *16%* | *84%* |
