# Supplementary material for: Beyond Neutralizing Antibody Levels: The Epitope Specificity of Antibodies Induced by National Institutes of Health Monovalent Dengue Virus Vaccines
Source: J Infect Dis. 2019 Mar 21;220(2):219–27. doi: 10.1093/infdis/jiz109 (PMC6581895; doi:10.1093/infdis/jiz109)
Supplement: jiz109_suppl_Supplementary_Table_S2 [file jiz109_suppl_supplementary_table_s2.docx]

**Supplementary Table S2: Viruses used for characterization**

| Virus | Strain | Backbone | Epitope | Epitope Donor |
| --- | --- | --- | --- | --- |
| DENV-1 | West Pac ‘74 |  |  |  |
| DENV-2 | S16803 |  |  |  |
| DENV-3 | UNC3001 Sri Lanka ‘89 |  |  |  |
| DENV-3’ | CH-53489 |  |  |  |
| DENV-4 | D4ic Sri Lanka ‘92 |  |  |  |
| DENV-4’ | TVP-360 |  |  |  |
| DENV2/1 |  | DENV-2 S16803 | 1F4 | DENV-1 West Pac ‘74 |
| DENV3/1 |  | DENV-3 UNC3001 | 1F4, 14C10 | DENV-1 West Pac ‘74 |
| DENV4/2 |  | DENV-4 D4ic | EDIII | DENV-2 S16803 |
| DENV4/3 |  | DENV-4 D4ic | 5J7 | DENV-3 UNC3001 |
